# Coinfection With Severe Fever With Thrombocytopenia Syndrome and Scrub Typhus in Korea

**DOI:** 10.1093/ofid/ofad377

**Published:** 2023-10-17

**Authors:** Shilpa Chatterjee, Choon-Me Kim, Dong-Min Kim, Jun-Won Seo, Da Young Kim, Na-Ra Yun, Sook In Jung, Uh Jin Kim, Seong Eun Kim, Hyun ah Kim, Eu Suk Kim, Jian Hur, Young Keun Kim, Hye Won Jeong, Jung Yeon Heo, Dong Sik Jung, Hyungdon Lee, Sun Hee Park, Yee Gyung Kwak, Sujin Lee, Rajendra Prasad Chatterjee

**Affiliations:** Department of Biomedical Science, College of Medicine, Chosun University, Gwangju, Republic of Korea; Premedical Science, College of Medicine, Chosun University, Gwangju, Republic of Korea; Department of Internal Medicine, College of Medicine, Chosun University, Gwangju, Republic of Korea; Department of Internal Medicine, College of Medicine, Chosun University, Gwangju, Republic of Korea; Department of Internal Medicine, College of Medicine, Chosun University, Gwangju, Republic of Korea; Department of Internal Medicine, College of Medicine, Chosun University, Gwangju, Republic of Korea; Department of Internal Medicine, Chonnam National University Medical School, Gwangju, Republic of Korea; Department of Internal Medicine, Chonnam National University Medical School, Gwangju, Republic of Korea; Department of Internal Medicine, Chonnam National University Medical School, Gwangju, Republic of Korea; Division of Infectious Diseases, Keimyung University Dongsan Hospital, Keimyung University School of Medicine, Daegu, Republic of Korea; Department of Internal Medicine, Seoul National University Bundang Hospital, Seoul National University College of Medicine, Seongnam, Republic of Korea; Department of Internal Medicine, Yeungnam University Medical Center, Daegu, Republic of Korea; Department of Internal Medicine, Wonju College of Medicine, Yonsei University, Wonju, Republic of Korea; Department of Internal Medicine, College of Medicine, Chungbuk National University, Cheongju, Republic of Korea; Department of Infectious Diseases, School of Medicine, Ajou University, Suwon, Republic of Korea; Department of Internal Medicine, College of Medicine, Dong-A University, Busan, Republic of Korea; Department of Internal Medicine, Chuncheon Sacred Heart Hospital, College of Medicine, Hallym University, Chuncheon, Republic of Korea; Division of Infectious Diseases, Department of Internal Medicine, College of Medicine, Catholic University of Korea, Seoul, Republic of Korea; Department of Internal Medicine, Inje University Ilsan Paik Hospital, Goyang, Republic of Korea; Department of Internal Medicine, College of Medicine, Pusan National University, Yangsan, Republic of Korea; National Institute of Cholera and Enteric Diseases, Indian Council of Medical Research, Kolkata, India

**Keywords:** coinfection, phylogenetic tree, polymerase chain reaction, scrub typhus, severe fever with thrombocytopenia syndrome

## Abstract

**Background:**

Scrub typhus and severe fever with thrombocytopenia syndrome (SFTS) are the 2 most common tick-borne infectious diseases in Korea. Every year, an increasing number of cases are reported, which is a public health concern. Therefore, we aimed to investigate the prevalence of SFTS–scrub typhus coinfection in patients with SFTS.

**Methods:**

Clinical samples were collected from 129 patients with SFTS. One-step reverse-transcription polymerase chain reaction (PCR) was performed to identify the SFTS virus (SFTSV), and real-time PCR followed by nested PCR was performed to detect the *Orientia tsutsugamushi* gene for scrub typhus. Phylogenetic analysis was conducted to confirm the evolutionary relationships among different species.

**Results:**

Among 129 SFTS cases, 2 patients with SFTSV were positive for *O. tsutsugamushi* with a prevalence of coinfection of 1.6% (95% confidence interval, .001–.06). Phylogenetic analysis confirmed these as *O. tsutsugamushi* strain Boryong.

**Conclusions:**

Our study found that 1.6% of patients were coinfected with SFTS and scrub typhus infection. We believe that this information will add a new dimension to clinical diagnosis, which should be considered for better public health management. Further research is needed to better understand the ecological transmission dynamics and geographical distribution of SFTSV and *O. tsutsugamushi* in endemic countries.

## Teaser

Clinical samples were collected from 129 patients with severe fever with thrombocytopenia syndrome (SFTS) to determine SFTS–scrub typhus coinfection prevalence in South Korea. Two patients were coinfected with the SFTS virus and *Orientia tsutsugamushi*.

Severe fever with thrombocytopenia syndrome (SFTS) is an emerging viral hemorrhagic fever caused by the SFTS virus (SFTSV) of the genus *Banyangvirus* and family Phenuiviridae and has a high mortality rate of 16.7% [1]. SFTS is characterized by fever, thrombocytopenia, gastrointestinal symptoms, and leukopenia [2]. *Haemaphysalis longicornis* and *Rhipicephalus microplus* ticks are the major vectors for the transmission of SFTSV to humans, and *H. longicornis* ticks are widespread in South Korea [2]. Additionally, SFTSV can be transmitted from dogs or cats to humans [3]. Furthermore, human-to-human and nosocomial transmission have also been reported in patients with SFTS [3]. Since the disease was discovered in China in 2011, cases have been reported in South Korea (2013) [4] and Japan (2012) [5]. Although SFTS can occur throughout the year, the high-risk tick bite season occurs between May and October.

Scrub typhus and SFTS are the 2 most common tick-borne infectious diseases in South Korea [2, 6]. Scrub typhus, a potentially fatal bacterial infection caused by *Orientia tsutsugamushi*, is predominantly spread by the bites of *Leptotrombidium* spp mites (Acari: Trombiculidae) [2]. Scrub typhus is prevalent in South Korea. Thousands of cases of scrub typhus are reported annually, mostly during the harvest season (October and November), with 10, 485 cases reported in 2013 [2].

Fever, myalgia, and gastrointestinal symptoms are clinical features shared by scrub typhus and SFTS. Additionally, the 2 diseases share risk factors, including a history of outdoor activity, farming, exposure to the countryside, chigger or tick bites, and an overlap in their geographic distribution [7]. Coinfections are a concern in clinical practice. In South Korea, individuals who are febrile and have a history of insect bites are typically thought to have scrub typhus and are treated with antibiotics such as doxycycline, azithromycin, or tetracycline, mostly in the early stages of the illness. We investigated the prevalence of SFTS–scrub typhus coinfection in patients with SFTS.

## METHODS

To investigate the prevalence of SFTSV and potential coinfection with *O. tsutsugamushi* in South Korea, we performed a multicenter clinical cohort study in which whole blood samples were collected prospectively, from 129 patients positive for SFTS from 14 hospitals between 2015 and mid-June 2022, and then stored for further diagnosis. The mean age of the patients was 69.4 years (range, 25–92 years). Samples were collected at a mean of 5.1 days after symptom onset. Among the 129 patients, 3 (2.3%) had eschars, and 25 (19.4%) deaths were reported. The highest number of suspected cases was identified in Jeolla province (64 [49.6%]), followed by Gyeonggi (33 [25.6%]), Gyongsang (25 [19.4%]), Gangwon (4 [3.1%]), and Chungcheong (3 [2.3%]) provinces.

Viral RNA was extracted from 300 µL of blood using the Viral Gene-spin RNA Extraction Kit (iNtRON Biotechnology, Seongnam, Korea) following the manufacturer's instructions. Molecular diagnosis of SFTSV infection was performed by detecting the partial medium (M) and small (S) segment genes of SFTSV using 1-step reverse-transcription polymerase chain reaction (PCR) (Supplementary Table 1) [8, 9]. Of the 129 patients, 122 (94.6%) were positive for SFTSV on PCR targeting the M segment, and 114 (88.4%) were positive for SFTSV on PCR targeting the S segment.

DNA was extracted from whole blood or serum samples using the QIAamp DNA Mini Kit (Qiagen, Hilden, Germany). The molecular diagnosis of *O. tsutsugamushi* was first performed using real-time PCR targeting the conserved hypothetical protein (tchA) of *O. tsutsugamushi* using an IRON-qPCR Tsutsugamushi real-time PCR kit (Bioneer, Daejeon, Korea). To confirm the results of quantitative PCR, nested PCR was performed targeting the 56-kDa antigen of *O. tsutsugamushi* using the INNOPLEX TSUTSU detection kit (iNtRON Biotechnology, Seoul, Korea) [10].

## RESULTS

**Figure 1.**
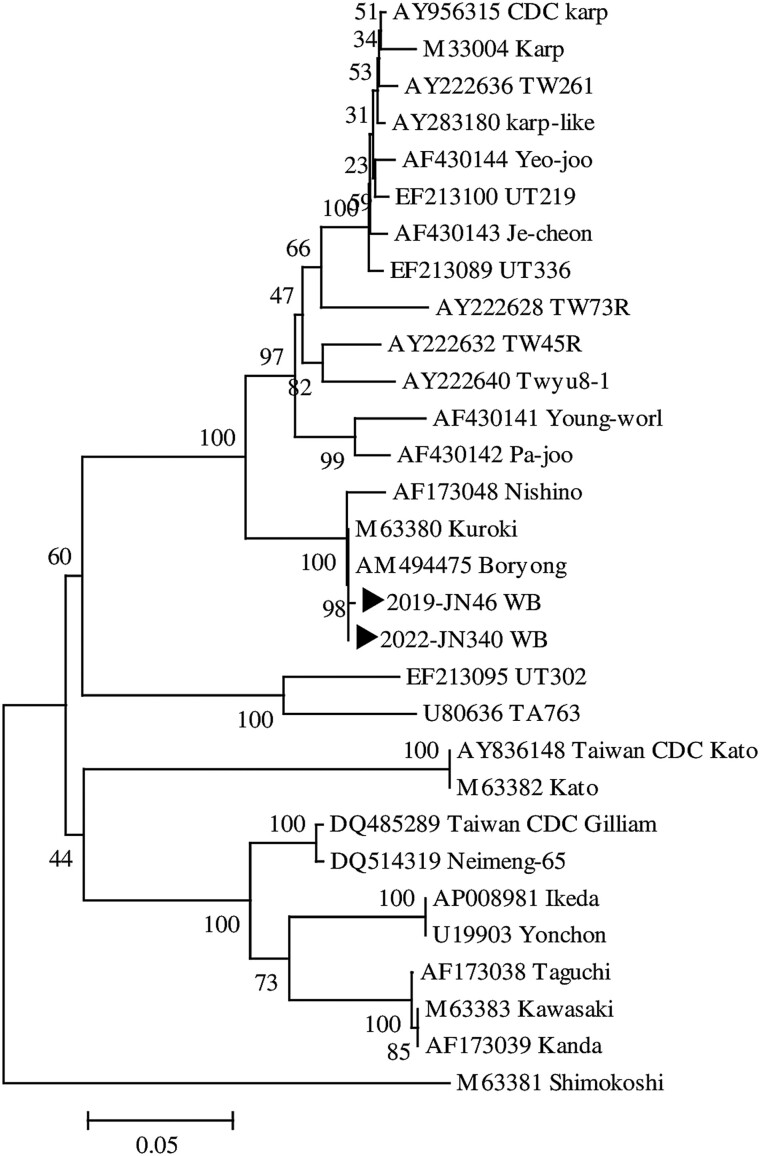
Phylogenetic tree constructed based on *Orientia tsutsugamushi* 56-kDa gene sequences (439 bp) from patients with scrub typhus in Korea (►) and 28 GenBank reference sequences.

Two of the 129 patients with SFTS tested positive for both target genes of *O. tsutsugamushi*. Both patients with coinfection were from the southern part of Jeolla province. One of the coinfected patients (patient 1) was a 79-year-old woman, and the other (patient 2) was a 64-year-old man. Detailed clinical characteristics and outcomes of these 2 patients are shown in Table 1. Patient 1 was diagnosed with SFTS in November 2019 and presented with a skin rash and eschar. The immunofluorescence assay (IFA) showed that the immunoglobulin G (IgG) and immunoglobulin M (IgM) antibody titers for *O. tsutsugamushi* were 1:32 and 1:64, respectively. Patient 2 had no skin rash or eschar. The IFA showed that both the IgG and IgM antibody titers for *O. tsutsugamushi* were 1:16. Both of the coinfected patients presented with fever, but only 1 of them had eschar and skin rash, which made the clinical differentiation of coinfection or single infection more difficult. The prevalence of coinfection was 1.6% (2/129; 95% confidence interval, .001–.06). Phylogenetic analysis was performed targeting *O. tsutsugamushi* 56-kDa gene sequences (475 bp) with ClustalX, and phylogenetic trees were constructed using the neighbor-joining method. The phylogenetic tree revealed that the 2 strains identified in this study clustered with the Boryong strain isolated in Seoul, Korea (Figure 1).

**Table 1.** Clinical Characteristics and Outcomes of 2 Coinfected Patients

Characteristic	Patient 1	Patient 2
Age, year	82	64
Sex	Female	Male
Comorbidities	DM, CVA	Chronic liver disease
Geographical location in Korea	Jeolla province	Jeolla province
Onset of illness to admission, day	8	0
APACHE II score	7	NA
Symptoms and signs		
Fever	Yes	Yes
Headache	None	None
Myalgia	None	None
Nausea/vomiting	None	None
Arthralgia	None	None
Regional lymphadenopathy	None	None
Abdominal pain	None	None
Altered mentality	None	Yes
Skin presentation		
Eschar	Yes	None
Skin rash	Yes	None
Laboratory findings		
WBC count, cells/μL	7200	3700
Leukopenia (<4000 cells/μL)	None	Yes
Hemoglobin, g/dL	11.4	14
Anemia (<11 g/dL)	None	None
Platelet count, ×1000 platelets/μL	55	162
Thrombocytopenia (<150 000 cells/μL)	Yes	None
Severe thrombocytopenia (<50 000 cells/μL)	None	None
C-reactive protein, mg/dL	10.72	0.94
AST, IU/L	72	50
ALT, IU/L	34	47
Total bilirubin, mg/dL	0.55	0.46
LDH, IU/L	710	887
Clinical course		
ICU admission	None	None
Mechanical ventilation	None	None
Hospital stay, day	13	11
Death	None	None
*Orientia tsutsugamushi* IFA titer (IgG/IgM)	1:32/1:64	1:16/1:16
PCR for *O. tsutsugamushi*	Positive	Positive
PCR for SFTSV	Positive	Positive

Abbreviations: ALT, alanine aminotransferase; APACHE II, Acute Physiology and Chronic Health Evaluation; AST, aspartate aminotransferase; CVA, cerebrovascular accident; DM, diabetes mellitus; ICU, intensive care unit; IFA, immunofluorescence assay; IgG, immunoglobulin G; IgM, immunoglobulin M; LDH, lactate dehydrogenase; NA, not applicable; PCR, polymerase chain reaction; SFTSV, severe fever with thrombocytopenia syndrome virus; WBC, white blood cell.

**Figure 2.**
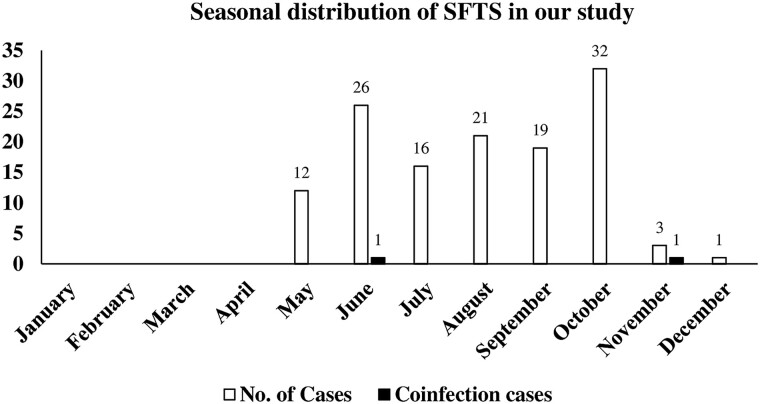
Seasonal distribution of severe fever with thrombocytopenia syndrome (SFTS) cases identified in our study between 2015 and 2022.

Moreover, the seasonal distribution of SFTS cases in our study indicated a high prevalence during the spring and fall seasons (June to October) (Figure 2). However, a few cases were observed in November and December (Figure 2).

## DISCUSSION

To date, very few cases of SFTS and scrub typhus coinfection have been reported. A study by Wi et al. [2] also suggested that SFTSV and *O. tsutsugamushi* coinfection may occur in South Korea. They reported a 41% coinfection prevalence, and antibody titer was determined using a commercial immunochromatography kit, which is less sensitive and specific than other gold-standard tests such as IFA or PCR.

The involvement of different vectors in SFTS and scrub typhus suggests that the true prevalence of coinfection is likely to be <41%. Subsequently, Yoo et al. reported a coinfection of SFTS and scrub typhus based on molecular analysis and observed the involvement of different vectors for each disease [11]. Although serological assays based on IFA are the gold standard for the detection of *O. tsutsugamushi*, they pose significant drawbacks in terms of the requirement of paired sera, higher antigenic diversity among serotypes, and standard antibody cutoff titer values to determine the true prevalence in endemic regions [12]. Furthermore, it is challenging to eliminate the possibility of past or recent infection in cases with a single high titer of scrub typhus antibody [12]. Therefore, to identify the true prevalence in a larger study population, we performed a multicenter clinical cohort study and systematically investigated the rate of coinfection based on molecular diagnostic methods.

The seasonal distribution of SFTS cases in our study indicated a high prevalence during the spring and fall seasons (June to October), which is very similar to the overall prevalence patterns reported by the Korea Center for Disease Control and Prevention and the National Notifiable Disease Surveillance System in South Korea during this time period [13]. However, only a few cases were observed in November and December. We believe that this information will add a new dimension to clinical diagnosis, which should be considered for better public health management.

SFTS and scrub typhus have similar signs and symptoms; however, SFTS has a higher fatality rate than scrub typhus [14]. Furthermore, scrub typhus tends to present with more diagnostically important clinical manifestations such as maculopapular skin rashes and eschar formation, which are distinct from those in SFTS and have diagnostic value for clinicians [15]. Therefore, the absence of an eschar or skin rash poses a challenge in the diagnosis of scrub typhus. In our study, only 1 of the 2 coinfected patients showed maculopapular rash and eschar formation. Therefore, it is crucial to consider SFTS and scrub typhus coinfection in patients detected with SFTSV. Moreover, further detailed research is required on the different vectors implicated in the spread of SFTS.

## CONCLUSIONS

This study indicated that 1.6% of patients had SFTS–scrub typhus coinfection. Therefore, a better understanding of the ecological transmission dynamics and geographical distribution of SFTSV and *O. tsutsugamushi* in endemic countries is required. Moreover, it is necessary to conduct ongoing surveillance of patients with SFTS to report in-depth clinical symptoms and related viral genotypes that are common in the region. In addition, improved clinical procedures and outcomes are required in areas where multiple tick-borne and mite-borne infections are common. However, further research is required to determine whether coinfection is caused by bites from different vectors or a common vector. In clinical setting, scrub typhus can be easily treated with doxycycline, azithromycin, or tetracycline antibiotics. Moreover, in regions where both diseases are common, empirical doxycycline treatment may be necessary until coinfection with scrub typhus in SFTS patients is eliminated. Additionally, more reliable differential diagnostic approaches are warranted to detect SFTS and scrub typhus coinfection in clinical settings.

## Supplementary Material

ofad377_Supplementary_Data

## Supplementary Data

Supplementary materials are available at *Open Forum Infectious Diseases* online. Consisting of data provided by the authors to benefit the reader, the posted materials are not copyedited and are the sole responsibility of the authors, so questions or comments should be addressed to the corresponding author.

## Notes

***Author contributions.*** Concept and design: D.-M. K. Acquisition, analysis, or interpretation of data: All authors. Drafting of the manuscript: S. C. Critical revision of the manuscript: S. C., C.-M. K., D.-M. K., and R. P. C. Administrative, technical, or material support: Y. G. K., S. L., and R. P. C. Supervision: D.-M. K. All authors have read and agreed to the published version of the manuscript.

***Data availability.*** Data and materials are available from the corresponding author on reasonable request.

***Patient consent.*** The study was approved by the Ethics in Human Research Committee of Chosun University Hospital (institutional review board numbers 2017-10-012 and 2021-10-001) as the central coordinating center, and written informed consent was provided by all participants.

***Financial support.*** This work was supported by a research fund from Chosun University, 2022.
