# Supplementary material for: Coinfection With Severe Fever With Thrombocytopenia Syndrome and Scrub Typhus in Korea
Source: Open Forum Infect Dis. 2023 Oct 17;10(10):ofad377. doi: 10.1093/ofid/ofad377 (PMC10580145; doi:10.1093/ofid/ofad377)
Supplement: ofad377_Supplementary_Data [file ofad377_supplementary_data.docx]

**Supplementary Table S1. Oligonucleotide primers used to detect the molecular target in SFTS virus**

| **Target** | **Primer** | **Nucleotide sequence (5′–3′)** | **Fragment length (bp)** | **Reference** |
| --- | --- | --- | --- | --- |
| M segment | SFTS-M 1st-F | TCATCCTGACYTATTYTGCAATWG | 640 | [7] |
|  | SFTS-M 1st-R | TAAGTYACACTCACACCCTTGAA |  |  |
|  | SFTS-F(=MF3) | GATGAGATGGTCCATGCTGATTCTAA | 560 |  |
|  | SFTS-R(=MR2) | CTCATGGGGTGGAATGTCCTCAC |  |  |
| S segment | SFTS-S-NP-2F | CATCATTGTCTTTGCCCTGA | 461 | [8] |
|  | SFTS-S-NP-2R | AGAAGACAGAGTTCACAGCA |  |  |
|  | SFTS-S-N2F | AAYAAGATCGTCAAGGCATCA | 346 |  |
|  | SFTS-S-N2R | TAGTCTTGGTGAAGGCATCTT |  |  |
